# Antagonist *Xist* and *Tsix* co-transcription during mouse oogenesis and maternal *Xist* expression during pre-implantation development calls into question the nature of the maternal imprint on the X chromosome

**DOI:** 10.1080/15592294.2015.1081327

**Published:** 2015-08-12

**Authors:** Jane Lynda Deuve, Amélie Bonnet-Garnier, Nathalie Beaujean, Philip Avner, Céline Morey

**Affiliations:** 1 Laboratoire de Génétique Moléculaire Murine; Institut Pasteur; Paris, France; 2 INRA; UMR1198 Biologie du Développement et Reproduction; Jouy-en-Josas, France

**Keywords:** imprinting, long non-coding RNAs, mouse oogenesis, mouse pre-implantation development, single-cell analysis, transcription, X-inactivation

## Abstract

During the first divisions of the female mouse embryo, the paternal X-chromosome is coated by *Xist* non-coding RNA and gradually silenced. This imprinted X-inactivation principally results from the apposition, during oocyte growth, of an imprint on the X-inactivation master control region: the X-inactivation center (*Xic*). This maternal imprint of yet unknown nature is thought to prevent *Xist* upregulation from the maternal X (X^M^) during early female development. In order to provide further insight into the X^M^ imprinting mechanism, we applied single-cell approaches to oocytes and pre-implantation embryos at different stages of development to analyze the expression of candidate genes within the *Xic*. We show that, unlike the situation pertaining in most other cellular contexts, in early-growing oocytes, *Xist* and *Tsix* sense and antisense transcription occur simultaneously from the same chromosome. Additionally, during early development, *Xist* appears to be transiently transcribed from the X^M^ in some blastomeres of late 2-cell embryos concomitant with the general activation of the genome indicating that X^M^ imprinting does not completely suppress maternal *Xist* transcription during embryo cleavage stages. These unexpected transcriptional regulations of the *Xist* locus call for a re-evaluation of the early functioning of the maternal imprint on the X-chromosome and suggest that *Xist*/*Tsix* antagonist transcriptional activities may participate in imprinting the maternal locus as described at other loci subject to parental imprinting.

## Introduction

The paternal and maternal genomes are not fully equivalent. In mammals, differences include the presence of parent-of-origin specific marks or “imprints,” which lead to monoallelic expression of either the maternally-inherited or paternally-inherited alleles of imprinted genes in embryonic and/or adult tissues. In female mice, an extreme example of imprinting is represented by the inactivation of the paternal X chromosome (X^P^), which characterizes extra-embryonic tissues, as opposed to embryonic and adult tissues, which display a random inactivation of the X^P^ or of the maternal X (X^M^), leading to tissues that are mosaic for the expression of X-linked genes. Imprinted X-chromosome inactivation (I-XCI) is first established at the 4-cell stage and manifests itself as overexpression of the *Xist* gene from the X^P^.^1-6^ *Cis*-coating of the X^P^ by *Xist* ncRNA, recruitment of chromatin remodelers, and heterochromatinization of the chromosome subsequently maintain the silent state of the inactive X in extra-embryonic derivatives of both the placenta and the yolk sac^7-9^ (see also^10^ for a review of X-inactivation mechanisms). In contrast, the X^P^ is reactivated in the embryonic lineage to allow the establishment of random X-inactivation in the epiblast of the late blastocyst.

**Figure 1.**
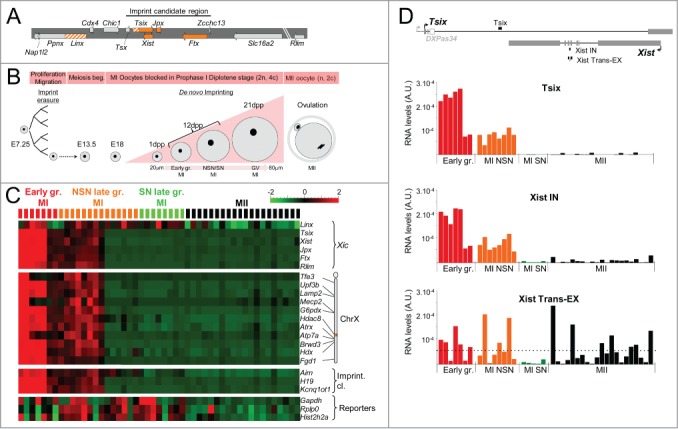
High transcriptional activities within the *Xic* in early-growing oocytes. (**A**) *Xic* map showing the non-coding genes in orange, the non-coding transcription units in hatched orange, and the coding genes in gray. The imprint candidate region described in^16^ is indicated. (**B**) Diagram representing the main phases of oogenesis. As early as E13.5, meiosis starts in primordial germ cells. Oocytes are arrested in prophase I at E18 (MI oocytes). Shortly after the birth of female mice, the primary oocytes are incorporated into primordial follicles. Upon follicle recruitment, such primary oocytes enter a growth phase to become fully-grown, GV-stage oocytes reaching prophase I (2n chromosomes, 4c chromatids). At puberty, the induction of ovulation leads to the breakdown of the germinal vesicle (or nuclear membrane), the resumption of meiosis associated with expulsion of the first polar body until the second metaphase (MII oocytes). The mature oocyte (1n, 2c) is then ready to be fertilized. During oocyte growth phase, the volume of the oocyte increases to reach 4-5 times its initial size. At 12 dpp the oocyte population of the ovary consists in a mixture of early-growing and late-growing oocytes that have been identified using a size-based criterion.^41,67,68^ Ovaries from older (3 to 6-week-old) mice have been used to obtain late-growing oocytes. SN oocytes have been separated from NSN by Hoechst staining. (**C**) Heatmap showing the levels of primary transcripts both within the *Xic*, at several X-linked genes and at lncRNAs of imprinted gene clusters in a population of early-growing MI (7), NSN (14) and SN (8) late-growing MI and MII (20) oocytes (129Sv mouse strain). Intronic assays have been used to quantify the primary transcription of coding genes and of lncRNAs when applicable (see **Table S1** for primer sequence). For each gene, absolute pre-RNA levels have been normalized by the mean and by the variance across the cell population. The levels of primary transcripts in early-growing oocytes are significantly different from corresponding RNA levels in late-growing MI and MII oocytes (t-test q-value < 10^−3^). Steady-state levels of 3 housekeeping RNAs (*Gapdh, Rplp0*, and *Hist2h2a*), measured using exonic assays, are shown as a control of RNA quality. See also hierarchical clustering of expression profiles and oocyte quality controls in **Figure S2**. (**D**) Histograms showing the absolute RNA levels measured using single-cell RT-qPCR at the indicated position in oocytes at different stages. The horizontal dotted line on the histogram marks the average level of spliced *Xist* RNA on the inactive X-chromosome in female somatic cells.^51,69^ Above the histograms, the map shows the reciprocal structures of the *Xist* and *Tsix* transcripts. The majority of *Tsix* transcription initiates upstream of the *DXPas34* minisatellite.^19,27,56^ The positions of the RT-qPCR assays used to detect *Tsix* transcription (*Tsix*) and primary/spliced *Xist* transcripts (*Xist IN*/*Xist Trans-EX*) are shown as solid bars above and underneath the map respectively. Exons: solid gray boxes; *DXPas34*: open gray box.

The nature of the imprint(s) on the X^P^ and/or on the X^M^ responsible for this I-XCI remains largely unknown. In the case of a paternal imprint that would predispose the X^P^ to inactivation, this mark would need to be rather labile since, in X^P^O females, the single X chromosome remains active in extra-embryonic tissues.^11^ In contrast, the existence of a robust maternal imprint protecting the X^M^ from inactivation is supported by pioneering studies using parthenogenetic/gynogenetic embryos (2 maternally derived pronuclei) or X^M^X^M^X^P^ and X^M^X^M^Y embryos, which show a delay in the establishment of XCI during pre-implantation development in the first case,^1,4^ and an absence of I-XCI in extra-embryonic tissues associated with placental defects in the second case.^1,5,12-15^ Such a maternal imprint must be located, at least in part, within the genomic span of a transgene that has been shown to reproduce accurate I-XCI during pre-implantation development when inserted as a single-copy on an autosome.^16^ The 210-kb candidate interval extends over part of the X-inactivation center (*Xic*) and includes—among others—the *Xist* gene,^17,18^ its *cis*-repressor, the *Tsix* antisense ncRNA,^19,20^ and 2 other ncRNAs, *Ftx* and *Jpx*,^21^ which are thought to positively regulate *Xist* expression^22,23^ (**Fig. 1A**, for review see^24^).

Mutations of both *Xist* and *Tsix* result in abnormal placental development associated with aberrant, parent-of-origin specific I-XCI profiles, which are consistent with the repression of *Xist* by *Tsix* on the X^M^ of extra-embryonic tissues.^25-27^ However, the initial repression of the maternal *Xist* allele during pre-implantation development is likely independent of *Tsix*, since this antisense transcript is not transcribed during embryo cleavage stages.^27^ At these early stages, a crucial factor implicated in the paternal activation of *Xist* is the ubiquitin ligase RLIM (encoded by the X-linked *Rnf12/Rlim* gene), as suggested by the lack of *Xist* coating in female embryos lacking RLIM.^28^ Based on these results, one of the current hypotheses proposes that the X^M^ imprint is established in the female germline and protects the maternal *Xist* allele from subsequent activation by RLIM at the time of zygotic gene activation.

During early post-implantation development (E7.25), female germ cells originating from the epiblast proliferate and migrate to the genital ridge. Around E10.5, the primordial germ cells colonizing the gonads are subject to global DNA demethylation and extensive histone modifications associated with global genome resetting^29,30^ (**Fig. 1B**). At this time, the inactive X undergoes global reactivation.^31,32^ Later, shortly after birth, primary oocytes enter a growth phase to become fully-grown, germinal vesicle (GV)-stage oocytes (2n chromosomes, 4c chromatids). This growth phase is characterized by the onset of strong transcriptional activity, increase of oocyte size, accumulation of maternal transcripts and proteins, and by the establishment of maternal autosomal imprints via the progressive methylation of differentially methylated regions (DMRs)(for review see^33-35^ and references therein). At the end of the growth phase, transcriptional arrest is associated with a drastic chromatin re-organization characterized by the formation of a ring of heterochromatin around the nucleolus.^36-38^ The transition from “not surrounded nucleolus” (NSN) to “surrounded nucleolus” (SN) nuclear organization is thought to accompany the acquisition of fertilization competency.^39,40^ At puberty, after cyclic hormonal stimulation, the GV breaks down, meiosis I resumes, and ovulation occurs.

Experiments exploiting nuclear transfers of GV from early oocytes into enucleated fully-grown oocytes have restricted the time window of X^M^ imprinting to the oocyte growth and final maturation stages.^41,42^ Intriguingly, no DMR-like element has been identified within the *Xic* up until now. Additionally, mutant embryos derived from oocytes that have been depleted either for the maintenance DNA methyltransferase DNMT1 or for the *de novo* DNA methyltransferases DNMT3A and DNMT3B during the oocyte growth develop normally at least until the blastocyst stage without any noticeable defects in the establishment of I-XCI.^43,44^ This suggests that the X^M^-imprint is unlikely to involve DNA methylation during cleavage stages. An alternative appealing hypothesis proposes that transcription itself participates in the X^M^-imprinting mechanism through the creation of a specific chromatin environment, which could allow imprint apposition. Such a mechanism has been described at the *Gnas* or at the PWS/AS (Prader-Willi/Angelman syndromes) imprinted loci, where transcription through DMRs during oogenesis is thought to promote the formation of an open chromatin structure allowing the imprinting complex access to DMRs.^45,46^ Similarly, transcription across imprint control regions is suspected to play a crucial role in the recruitment of specific histone modifications during spermatogenesis thereby participating in the establishment of paternal imprints.^47^

Following on the hypothesis of the involvement of transcription in the establishment of the maternal imprint on the X-chromosome, we have analyzed, using single-cell RT-qPCR and RNA-FISH approaches, the expression of candidate *Xic* genes and of X-linked genes in oocytes at different stages of folliculogenesis. Unexpectedly, all the *Xic* genes that we tested—including the *Xist* gene— appear heavily transcribed in early-growing oocytes. Strikingly, the activation of *Xist* takes place simultaneously with *Tsix* transcription, indicating that the antagonist relationship that exists between these 2 *Xic* actors in other cellular contexts cannot be operating in the growing oocyte. Maternal *Xist*, but not *Tsix* transcription, is then re-initiated, transiently, in late 2-cell embryos just before paternal *Xist* upregulation and X^P^ coating. This furtive maternal transcription may represent a “leakage” of the maternal *Xist* promoter that is selectively suppressed at later developmental stages. Alternatively and more interestingly, this permissive maternal expression may represent a backup system that is activated in abnormal developmental contexts, for example in parthenogenote and gynogenote embryos, to restore X-inactivation from the early morula stage onwards.^1,4^

## Material and Methods

### Animals

Animals were housed in the Institut Pasteur animal facilities accredited by the French Ministry of Agriculture to perform experiments on live mice (accreditation # 75-15-01, issued on 06/09/2013), in appliance of the French and European regulations on care and protection of the Laboratory Animals (EC Directive 86/609, French Law 2001-486 issued on June 6, 2001).

Protocols were approved by the veterinary staff of the Institut Pasteur animal facility and were performed in compliance with the NIH Animal Welfare Insurance #A5476-01 issued on 02/07/2007.

### Oocyte and embryo collection

Early-growing and late-growing GV-intact oocytes were collected without any hormonal stimulation, from freshly isolated ovaries obtained from 12-day old 129.Tgn.GFPX4,^48^ 129Sv, or C57BL/6 mice. Fully grown GV-intact oocytes NSN and SN were collected from a non-stimulated 6-week old 129Sv mouse. Ovaries were placed in M2 media (Sigma Aldrich) supplemented with dibutyryl cyclic AMP (dbcAMP, 0.1 mg/mL, Sigma Aldrich). Oocytes were released in the medium by puncturing the ovaries, and follicular cells were mechanically removed by mouth glass pipetting. In order to distinguish SN from NSN oocytes, the cells were incubated in the presence of Hoechst dye (2 μg/mL), as described previously.^40^ Mature MII oocytes were collected from 3-week old hormone stimulated mice: mice received an intraperitoneal (i.p.) injection of 7.5 IU pregnant mare serum gonadotropin (PMSG, Sigma) for 129Sv mice or of 5 IU for Pwk/PhJ mice, followed 46 h later by an i.p. injection of 5 IU human chorionic gonadotropin (hCG, Sigma). In order to obtain embryos, superovulated 129Sv mice were mated with Pwk/PhJ males or reciprocally. The same conditions were used to generate F1 embryos from C57BL/6 × 129Sv. The oviducts were flushed with M2 medium 24, 37, 47, 52, 62, or 74 h post hCG to collect zygotes, early 2-cell, late 2-cell, 4-cell, 8-cell, and morulae, respectively. After zona pellucida removal using acidic tyrode's solution (Sigma Aldrich), single blastomeres were dissociated mechanically using a glass pipet in trypsin solution. Importantly, all the embryos analyzed in this study were freshly collected and did not undergo any period of culture *in vitro*.

### RNA quantifications

#### Double-strand priming

Single-cell gene expression analyses were performed as described in Guo et al.^49^ and Rugg-Gunn et al.^50^ and as recommended by Fluidigm. Individual oocytes, whole embryos at different stages, or single blastomeres were distributed into wells containing 5 μl of CellsDirect resuspension buffer (Invitrogen Life Technologies). Gene-specific reverse transcription was performed using a mix of forward and reverse primers. This was followed by a gene-specific pre-amplification step, which consisted of 20 cycles using the same primer mix as for the RT step to pre-amplify each gene simultaneously. Controls for the absence of PCR bias due to this pre-amplification step are shown in **Figure S1A and S1B**. Pre-amplification was followed by exonuclease I treatment (NEB) and qPCR using nested allele-specific primers (when required) was performed on the BioMark thermocycler (Fluidigm). Raw efficiencies of each PCR assay were measured on control DNA or cDNA within each experiment. The allele-specificity of allelic primer pairs was controlled as previously described.^51^ Control for the absence of PCR bias of allelic ratios during the pre-amplification phase is shown in **Figure S1C**. Transcript levels were extrapolated using the raw PCR efficiencies allowing the direct comparison of different genes. Cells that fail to show any amplification, that are inherent to single-cell RT-qPCR analyses^49^ were identified using parallel quantifications of several control genes in all the single-cell RT-qPCR analyses presented here (3 housekeeping genes were systematically monitored: *Gapdh, Rplp0*, and *Hist2h2a*). Only cells showing a significant expression for 2 of these control genes are shown in the manuscript figures. Detailed analyses, statistical tests, and hierarchical clustering have been performed using the Qlucore Omics Explorer 2.3 (QLUCORE Company). As recommended for single cell gene expression analyses that are designed to reveal the variability of expression levels from cell to cell, no standardization with reporter genes has been applied. The variability of RNA levels from one cell to the other reflects the gene expression heterogeneity within the cell population.

#### Strand-specific priming

For single-cell strand-specific RT-qPCR, the general protocol and controls are described in **Figure S5A**. Compared to the standard protocol of double-strand priming, a step of DNase I treatment was added and performed as recommended by the manufacturer (CellsDirect One-step qRT-PCR kit, Invitrogen Life Technologies). Forward or reverse primers (50 nM) each were used to prime the reverse transcription reaction.

Embryos sexing was performed using PCR assays specifically detecting repeated sequences on the Y chromosome (sexF1: 5′-TGGAAAATGAGGAAAACCACTCTGT-3′; sexR1: 5′-ACGGTGTGCTACACTTTGCG-3′; sexF2: 5′-ACCACACTGTTGAACATTGTCGA-3′; sexR2: 5′-TGTTGTAACTCCTTTCCATGCCA-3′). Allele-specific qPCR primers were controlled for specificity and efficiency as described in ^51^ (see **Table S1** for primer sequences).

### Sequential RNA-DNA-FISH

The procedure was adapted from.^16,52^ Briefly, after removal of the zona pellucida, oocytes or embryos were washed 3× in BSA-PBS (6 mg/mL), placed on Superfrost+ glass slides coated with Denhardt, air dried for 30 minutes at room temperature, permeabilized, and fixed on ice in PBS 1X containing PFA (1%), Tergitol (0.05%) and vanadyl ribonucleoside complex (NEB, 2 mM) for 5 min and then in 3% PFA PBS 1X for 10 min prior to storage in 70% ethanol at 4°C. For RNA-FISH, slides were progressively dehydrated, then hybridized at 37°C overnight with fluorescent Fosmid/BAC/plasmid probes: *Xist/Tsix* (G135P602114E8, WI1-2363H9); *Tsix* double-stranded probe (17E)^53^; *Rlim* (G135P605237C7, WI1-2704K12); *Linx* (G135P603710E1, WI1-146H23); *Kdm5c* (RP24-148H21); *Xic* (G135P67398B12, WI1-415N1) labeled by nick translation (Vysis kit, Molecular Probe); *Xist* specific fluorescent oligonucleotides (*Xist*^*sense*^ mix of CT*C AGT CTT ATA GGC TGA GT*G ATG GGC ACT G and of AT*A GGA CTG CAT GCA T*TA AGT GAA ACT CCA T at 1 μM each, for *Xist*^*sense2*^, sequences available upon request, Stellaris, Biosearch Technologies); *Tsix* specific fluorescent oligos (*Tsix*^*antisense*^, sequences available upon request, Stellaris, Biosearch Technologies) together with mouse *Cot-1* DNA (Invitrogen) and sonicated salmon sperm DNA (Invitrogen). Slides were washed 2× in 50% Formamide/2XSSC (pH7.4) at 37°C for 5 min, 1× in 2XSSC at 37°C for 5min and mounted in Vectashield + DAPI (Vector Laboratories). The efficiency and specificity of the *Xist* and *Tsix* fluorescent oligos were measured through hybridization of differentiated ES cells, in which the *Xist* nuclear domain was easily detected by the single-stranded probe. We noted, however, that the *Xist* signal appeared weaker than when using a larger, double-stranded probe. This may induce a slight underestimation of the number of *Xist* transcribing loci. For sequential DNA-FISH procedure, slides were treated with RNaseH (10 U/ml NEB) for 30 min at 37°C, washed in 2XSSC, denatured in 70% Formamide/2XSSC (pH 7.4) for 1 min 30 sec at 75°C, dipped in ice-cold 2XSSC and hybridized overnight at 42°C with a probe mixture denatured for 10 min at 75°C and containing, approximately, 100 ng of each spectrum-labeled fosmid probe together with 3 μg of mouse *Cot-1* DNA and 10 μg of sonicated salmon sperm DNA per 22×22 mm coverslip. Washing and mounting steps were as described for the RNA-FISH procedure. Z-stacks were captured (step = 0.2 μm) on a Zeiss Axioplan2 microscope equipped with a Hamamatsu orca-ER CCD camera and controlled by the Perkin Elmer acquisition software Volocity.

### Poly(A)^+^ RNA pull down

Approximately 1000 mature MII oocytes were collected from 3-week old hormone stimulated 129Sv mice. Total RNA from MII oocytes was mixed with *Drosophila* larvae carrier RNA and poly(A)^+^ RNAs were separated from poly(A)- RNAs on oligo(dT) Dynabeads (Invitrogen). Following reverse transcription, qPCRs for Taf11 (*Drosophila* control; F: TTT GCA TTA CAG GCT TGA CG; R: AAG GAA CTG GAG GAG GAG GA) for *Xist* and for *Rlim* (see primer sequences in **Table S1**) were performed on poly(A)^+^ and poly(A)- RNA fractions in parallel. RNAs from female embryonic stem cells (LF2 cell line) differentiated for 4 days served as positive control for the presence of *Xist* poly(A)^+^ RNAs.

## Results

### Global *Xic* transcription occurs in early-growing oocytes

In order to analyze the transcriptional activities associated with the *Xic* and with X-linked genes during oogenesis, we collected early-growing MI-oocytes (n = 7), late-growing MI oocytes (n = 22), and mature MII oocytes (n = 20) from 129Sv mice of various ages (**Fig. 1B**). Following classification of the chromatin conformation by Hoechst staining, we identified 14 NSN oocytes and 8 SN oocytes among the late-growing group. We then measured transcription activities using intronic RT-qPCR assays combined with the Biomark technology (Fluidigm), which allows for parallel quantification of several RNA targets from a given cell^49^ (see **Table S1** for primer sequences and see Material & Methods section and **Figure S1 and S2** for in-depth description of the technology and the controls that have been performed). We analyzed the transcription levels at the *Xist, Tsix, Jpx, Ftx, Linx*, and *Rlim* loci, at a selection of X-linked genes and, for reference, within the body of the lncRNAs *Airn, Kcnq1ot1*, and *H19* genes involved in the imprinted expression of the autosomal gene clusters *Igf2r, Kcnq1*, and *H19-Igf2*, respectively (**Fig. 1C**). Controls of ubiquitously expressed reporter genes and genes expressed during oogenesis were also included (**Fig. 1C**, **Fig. S2A** and **S2B**).

We observed much higher transcriptional activity for genes within the *Xic* in most early-growing oocytes compared to late-growing MI oocytes or to MII oocytes (**Fig. 1C** and see **Table S2** for raw quantification results). Two out of 7 oocytes classified as early-growing, however, showed low levels of primary transcripts overall and clustered with SN oocytes (**Figure S2C**), suggesting that these oocytes were in fact more advanced in their growth. About half of NSN-oocytes showed significant—yet reduced compared to earlier oocytes—transcription. In agreement with previous reports indicating that transcription activity is restricted to the growth phase of oogenesis, transcription could barely be detected in SN MI- or in MII-oocytes.^46,52^ We noted, however, that the transcription profile of the *Linx* locus appeared more heterogeneous than those of other loci. While most *Linx* noncoding transcription occurs in early-growing oocytes, significant levels of transcripts were observed in some transcriptionally silent NSN and SN oocytes. The significance of this result is unclear, since the characteristics of the *Linx* non-coding transcription and, notably, the respective contributions of transcriptional activity, of primary transcripts, and of alternative spliced isoforms to global transcription activity at this locus have yet to be fully established.^54^

Taken overall, these observations indicate that the component loci of the *Xic*, like most of the X-linked and autosomal loci we tested, undergo transcriptional activation in early-growing oocytes.

### *Xist* and *Tsix* are co-transcribed in early-growing MI oocytes and mature *Xist* RNAs accumulate in MI and MII oocytes

Intriguingly, *Xist* and *Tsix*, two sense and antisense transcriptions known to be mutually exclusive in most cellular contexts,^55^ seem to show a similar degree of transcriptional induction in early oocytes (**Fig. 1C**). Since the *Xist* gene is almost completely embedded within an intron of *Tsix*, the intronic PCR assay for *Xist* (*Xist IN*) theoretically also detects ongoing *Tsix* transcription (see map of the *Xist/Tsix* locus in **Fig. 1D**). In order to discriminate sense from antisense transcriptions within the body of the *Xist* gene, we reasoned that, in the case of an active transcription in the *Xist* orientation, we should observe a concomitant accumulation of spliced *Xist* RNAs. To test this hypothesis we used a trans-exonic PCR assay (Xist Trans-EX) surrounding Xist IN and compared the levels of *Xist* spliced transcripts measured with Xist Trans-EX, to RNA levels quantified with Xist IN and to the intensity of *Tsix* transcription outside of the *Xist* gene (Tsix assay) in oocytes at different stages (**Fig. 1D**). While Tsix and Xist IN assays gave very similar transcription profiles throughout oogenesis, we detected an accumulation of mature *Xist* transcripts in oocytes of various stages, with RNA levels reaching levels found in somatic cells subject to X-inactivation (**Fig. 1D**). Similarly high levels of *Xist* RNAs were measured in MII oocytes from different genetic backgrounds indicating that *Xist* RNA accumulation is not subject to important strain specific variation and is likely to be a universal feature of oogenesis (**Figure S3**). We noted however that the level of *Xist* mature transcripts appeared highly heterogeneous among MII oocytes. The reason for this is not clear but may be linked to variable fertilization competency characterizing MII oocytes.

In order to further characterize the transcription at the *Xist*/*Tsix* locus in growing oocytes, we designed additional PCR assays along the locus that were used to analyze a replication set of 15 freshly collected early-growing oocytes (**Figure S4**). All three *Tsix*-specific assays detected significant levels of *Tsix* molecules indicating that, most probably, *Tsix* transcription extends over the entire locus. Similarly, no significant difference was observed between *Xist* trans-exonic or between *Xist* intronic assays, suggesting that *Xist* is fully transcribed and spliced in growing oocytes (**Figure S4**). Using strand-specific RT-qPCR on another batch of 21 early-growing oocytes (see **Figure S5** and Material and Methods section for detailed protocol and controls), we could detect, using primers within *Xist* intron 1, transcription in the *Xist* orientation in the majority of oocytes. This *Xist* transcription was accompanied by a significant transcription in the *Tsix* orientation in ∼¼ of oocytes (**Figure S6**). Surprisingly, at this position, *Tsix* RNA levels appeared very reduced compared to *Xist* levels or to *Tsix* levels at a downstream position within *Tsix* exon 4 (Tsix-2 assay, **Figure S4**). This suggests that, as previously characterized in ES cells, *Tsix* transcription is less abundant within *Xist* span and that *Tsix* spliced forms are more stable than *Tsix* primary transcripts.^56^

These results indicate that *Xist* and *Tsix* are co-expressed in early-growing oocytes. Interestingly, similar sense and antisense co-transcriptions were observed at 2 other autosomal imprinted loci: *Airn/Igf2r* (**Figure S7**) and *Kcnq1ot1/Kcnq1* (see **Table S2**).

### *Xist* and *Tsix* are transcribed from the same chromatid in MI oocytes

The detection of major levels of transcription at the *Xist/Tsix* locus by RT-qPCR quantification led to 2 different questions: (1) Is the accumulation of mature *Xist* transcripts in MI oocytes associated with the formation of a *Xist* domain on the X chromosome, which could directly participate in the imprinting process? (2) Are *Xist* and *Tsix* transcripts produced from the same locus or from distinct X chromosomes and/or sister chromatids present in the early-growing oocyte? In order to address these questions we analyzed, by RNA-FISH, the distribution of *Xic* transcripts in the nucleus of oocytes at different stages.

**Figure 2:.**
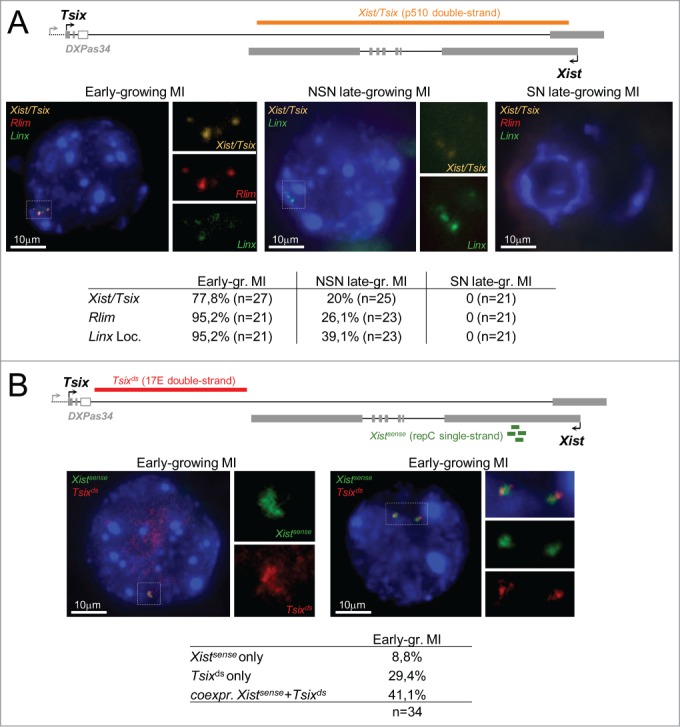
Transcription and nuclear organization of *Xic* transcripts in early-growing MI oocytes and in late-growing NSN and SN MI oocytes (129Sv). (**A**) Representative images showing the maximal projections of early-growing MI oocytes, and late growing MI oocytes showing either an NSN or an SN chromatin conformation after RNA-FISH for *Xist*/*Tsix*, for *Rlim* and for the *Linx* locus. Probes used for hybridization are indicated on each image. The position of the double-stranded probe detecting both *Xist* and *Tsix* (orange) is shown on the map above the pictures. Magnifications of the nuclear area around the signals are shown. The table underneath the pictures shows the percentage of oocyte showing an RNA-FISH signal at the indicated locus for each category of oocytes. (**B**) Transcription at the *Xist*/*Tsix* locus analyzed in RNA-FISH using a double-stranded *Tsix* specific probe (red) and a single-stranded *Xist* specific fluorescent oligonucleotide (green) located within *Xist* repeat C ^70^. Images of 2 different early-growing oocytes are shown together with magnifications of nuclear area around the signals. The position of the probes used in this panel is shown on the map above the pictures. The table underneath the RNA-FISH images indicates the percentage of early-growing oocytes showing *Xist* or *Tsix* transcription only, or showing simultaneous *Xist* and *Tsix* transcription from at least 1 chromatid.

In most early-growing oocytes, *Xist/Tsix, Rlim*, and *Linx* transcripts formed 3 to 4 pinpoint foci located in the same nuclear vicinity (**Fig. 2A**). Sequential DNA-FISH with *Xic* probes further indicated that these pinpoints co-localize to the endogenous gene loci and reflect ongoing *Xic* transcription from different X-chromatids (**Fig. S8A**). In later growing MI oocytes, while *Linx* was still transcribed in ∼40% of nuclei, *Rlim* transcription and transcription from the *Xist/Tsix* locus were detected in only 20 and 26% of the GVs in NSN oocytes, respectively (**Fig. 2A**). These results are consistent with the numbers of NSN oocytes in which primary RNAs for *Linx, Rlim*, and *Xist*/*Tsix* could be detected by RT-qPCR (see **Fig. 1C**). Of note, *Rlim* expression profile during oogenesis also supports a role for this gene in X^M^-imprinting.^28^ As expected for silent oogenic stages, no RNA-FISH signals could be detected at transcription sites in SN MI-oocytes or in MII-oocytes. Importantly, no accumulation of spliced *Xist* RNAs on the X-chromosomes was observed at any of the stages examined. One possible hypothesis to explain this result would be that the form of spliced *Xist* transcripts present in MII oocytes has a much reduced poly(A) tail compared to mature *Xist* RNAs found in adult cells, which may affect the ability of *Xist* RNAs to coat the X-chromosome in the context of the oocyte (**Figure S9**).

In order to address whether sense and antisense transcription at the *Xist/Tsix* locus originated from the same chromosome/chromatid, we performed double RNA-FISH on early-growing oocytes using a strand-specific oligonucleotide probe that specifically detects *Xist* transcripts and with a double-stranded probe lying 3′ to *Xist* within the *Tsix* 5′ end (**Fig. 2B**). We observed superimposed *Xist* and *Tsix* signals on 1 to 3 chromatids in ∼40% of the early-growing oocytes, a proportion slightly larger than the number of oocytes showing *Xist/Tsix* co-expression, as measured by strand-specific RT-qPCR (**Fig. S6B**). Since strand-specific single-cell RT-qPCR reflects the quantities of a given RNA molecule in a given cell at a given time and, therefore, depends on transcript stability while RNA-FISH detection only reflects, under our experimental conditions, ongoing transcription, it is possible that the above results indicate differences in the stability of *Tsix* ncRNAs compared to *Xist* nascent transcripts in this cellular context. For comparison, we performed the same RNA-FISH analysis on ES cells, which constitute the only known somatic cell type in which *Xist* and *Tsix* are co-transcribed, although *Xist* steady-state levels, in these cells, are dramatically reduced compared to *Tsix* levels.^56-59^ In these cells, only ∼5% of *Xist* loci showed simultaneous sense and antisense transcription (**Figure S8B**) indicating that *Xist* and *Tsix* co-transcription is more frequent in early-growing oocytes than in somatic cells.

To summarize, both our RNA-FISH and RT-qPCR results identify an accumulation of *Xist* spliced forms that do not coat the X-chromosomes in fully-grown MI and MII oocytes, suggesting either that these *Xist* RNA molecules are unable to associate with the X, potentially due to a short poly(A) tail, or that the X-chromosome structure is not accessible or, alternatively that some factors involved in the recruitment of *Xist* RNAs to the X-chromosome are not present in oocytes. Since *Xist* transcription in early-growing oocytes frequently occurs simultaneously with and from the same chromatid as antisense *Tsix* transcription, this strongly suggests that the repression of *Xist* by *Tsix* that is known to take place in other cellular contexts is less—or not—effective in early-growing oocytes.

### *Xist* is transiently transcribed from the X^M^ in late 2-cell embryos

**Figure 3.**
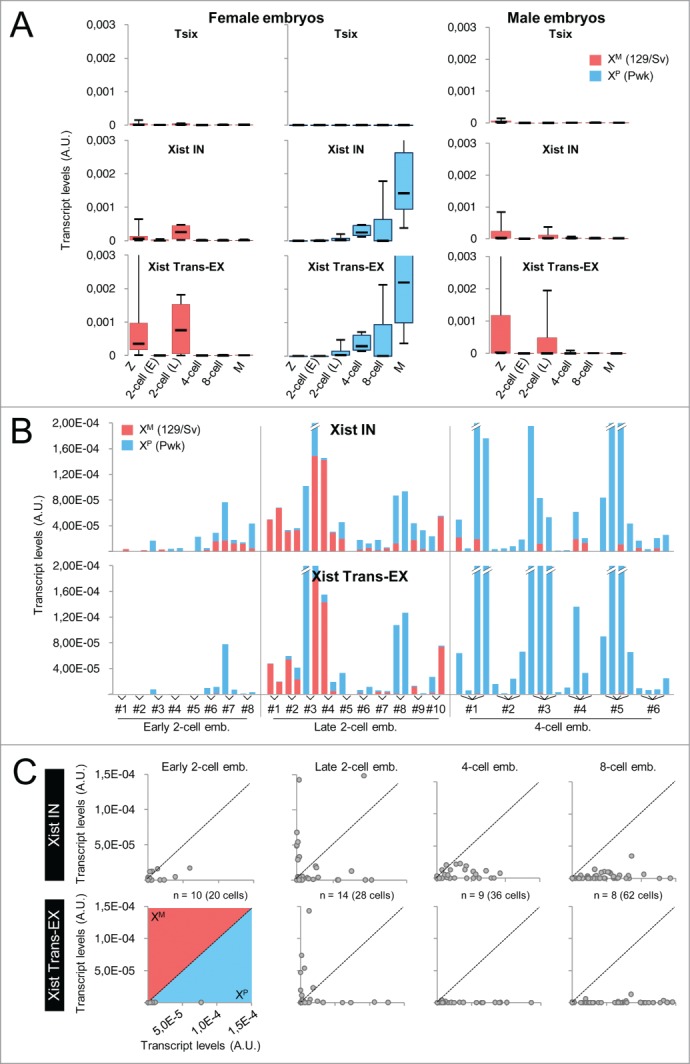
Allele-specific RT-qPCR analysis of transcription at the *Xist/Tsix* locus in 129Sv/Pwk pre-implantation embryos. (**A**) Box-plots showing the distribution of transcript levels in whole female and male embryos obtained from a 129Sv × Pwk cross assessed by RT-qPCR using the Biomark technology. Tsix, Xist IN, and Xist Trans-EX PCR assays are the same as in **Figure 1C** except that allelic assays have been used here (see **Table S1** for primer sequence). Z: zygotes (female, n = 10; male, n = 4); 2-cell (E): early 2-cell embryos (female, n = 5; male, n = 4); 2-cell (L): late 2-cell embryos (female, n = 4; male, n = 4); 4-cell: 4-cell embryos (female, n = 5; male, n = 6); 8-cell: 8-cell embryos (female, n = 7; male n = 10); M: morulae (female, n = 6; male, n = 7). See Materials and Methods section for embryo sexing. The levels of maternal *Xist* transcripts in late 2-cell embryos are significantly different from the levels of maternal *Xist* transcripts in early 2-cell or in 4-cell embryos in both male and female (*P* <0.05 by KS test). Only embryos showing significant expression of reporter housekeeping genes *Gapdh, Rplp0*, and *Hist2h2a* are shown. (**B**) Cumulative histograms showing the relative amounts of paternal (blue) and maternal (red) transcripts in dissociated cells of 129Sv/Pwk female embryos at the indicated stage assessed using single-cell allelic RT-qPCR. A representative selection of results is shown (see **Table S3** and panel C for complete results). Only embryos showing significant expression of reporter housekeeping genes *Gapdh, Rplp0*, and *Hist2h2a* in all blastomeres are shown. (**C**) Scatter-plots of expression levels from the paternal (x-axis) relative to the maternal (y-axis) X chromosome measured with the indicated RT-qPCR assay in individual cells of embryos at the indicated pre-implantation stage. Each dot represents a cell. A significant difference in *Xist* expression from the X^M^ is detected with both Xist IN and Xist trans-EX in cells of late 2-cell embryos as compared to cells of embryos at either earlier or later stages of development (*P* <0.05 by KS test). Only blastomeres showing significant expression of reporter housekeeping genes *Gapdh, Rplp0*, and *Hist2h2a* are shown.

*Xist* expression from the X^M^ in early-growing oocytes opens up the possibility that maternal *Xist* transcription also occurs during the first embryo cleavage stages. To test for this, we first performed allelic RT-qPCR quantifications of the *Xist* locus on whole 129Sv/Pwk heterozygous embryos at different pre-implantation stages (**Fig. 3A**, see also **Table S3** for raw quantification results and for RNA quantification of housekeeping control genes *Gapdh, Rplp0*, and *Hist2h2a*). As expected from previous reports, no *Tsix* expression could be detected during pre-implantation development either from the X^M^ or from the X^P^. In contrast, paternal transcription at the *Xist* locus appeared induced from the late 2-cell stage onwards, with *Xist* spliced transcripts progressively accumulating in the 4-cell female embryos. This kinetics is in agreement with previous reports of an initiation of X^P^ inactivation occurring around the 2 to 4-cell stage.^6,16^ More surprisingly, we also detected significant *Xist* expression from the X^M^ in the late 2-cell embryos of both sexes, which appeared to be turned off by the 4-cell stage (**Fig. 3A**). In order to address whether this *Xist* maternal transcription could be linked to the genetic background of the X^M^, we repeated our analysis on embryos of the reciprocal cross and obtained similar kinetics of maternal *Xist* induction at the late 2-cell stage specifically (**Fig. S10A**). We noted however that maternal *Xist* induction from an X chromosome of Pwk origin appeared reduced compared to transcription from a 129Sv X^M^ despite the fact that Pwk oocytes showed higher *Xist* levels than 129Sv oocytes (**Fig. S3**) indicating that the genetic background may influence the level of maternal *Xist* expression.

We next performed single cell RT-qPCR analysis of *Xist/Tsix* transcription to establish the actual number and proportion of blastomeres showing this maternal expression of *Xist*. While in individual cells of the early 2-cell female embryos, *Xist* expression was absent from the vast majority of cells, at the late 2-cell stage, we were able to detect an induction of maternal *Xist* in ∼½ embryos in which either one or both blastomeres appeared to produce both *Xist* primary and spliced transcripts (**Figs. 3B, 3C**, and **S10B**, see also **Table S3** for raw quantification results and for RNA quantification of housekeeping control genes *Gapdh, Rplp0*, and *Hist2h2a*). At the 4-cell stage, this maternal expression decreased drastically and *Xist* expression then became progressively restricted to the high levels of paternal transcription seen in cells of the 8-cell embryos (**Fig. 3C**). Male embryos also transcribed *Xist* at the late 2-cell stage in 2 out of 4 embryos (3 out of 8 blastomeres) (**Fig. S10C**).

**Figure 4.**
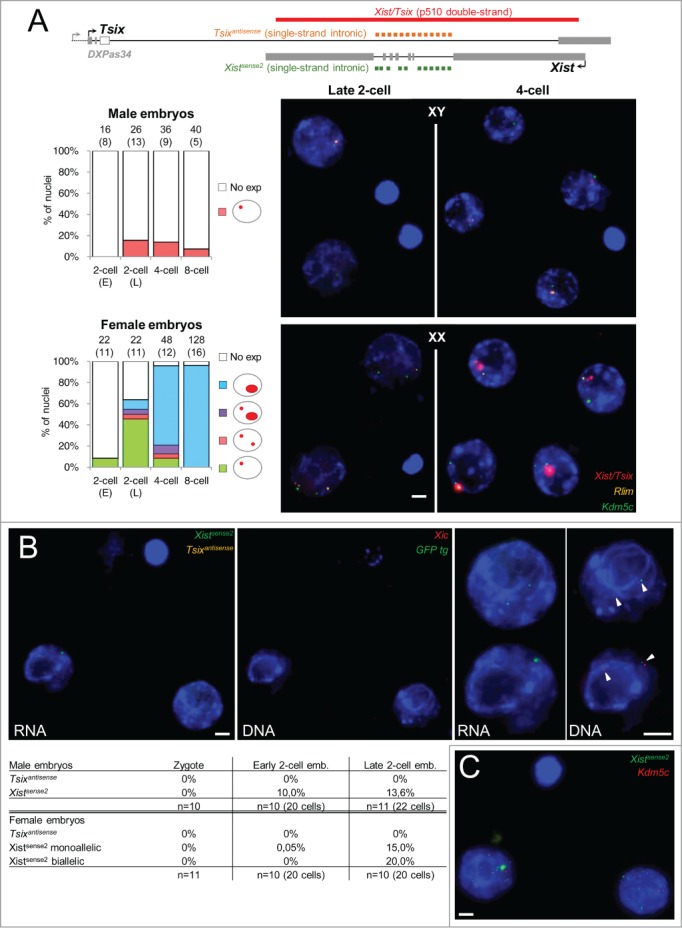
*In situ* transcription and nuclear organization of *Xist* ncRNAs during pre-implantation development. (**A**) Representative images showing the maximal projections of male and female late 2-cell and 4-cell embryos (129Sv/Pwk) after RNA-FISH for *Xist*/*Tsix*, for *Rlim* and for *Kdm5c*. Bar scale = 5 μm. Histograms on the left show the percentages of nuclei displaying the indicated RNA-FISH profile. The number of cells analyzed and, in brackets, the corresponding number of embryos is indicated above each column. Above, the map shows the position of RNA-FISH probes used in panel A and B. (**B**) Transcription at the *Xist*/*Tsix* locus analyzed in RNA-FISH using single-stranded *Tsix* specific fluorescent oligonucleotides (yellow, *Tsix*^*antisense*^) and single-stranded *Xist* specific fluorescent oligonucleotides (green, X*ist*^*sense2*^) located within *Xist* introns (see map in panel A). Representative images showing the maximal projections of a female late 2-cell embryo (129Sv/129Sv-GFP) after RNA-FISH (left) and after sequential DNA-FISH for the *Xic* (red) and for the GFP transgene (green) are shown. Magnifications of each nucleus are shown on the right of embryo images. The arrowheads indicate the location of the DNA-FISH signals. Bar scale = 5 μm. The table underneath the images indicates the percentages of zygotes, early 2-cell and late 2-cell embryos showing the indicated expression profile at the *Xist* locus. The table shows pooled results from C57BL/6 × 129Sv cross and from Pwk × 129Sv-GFP cross. No significant difference was observed between the 2 crosses. (**C**) Example of scattered *Xist* RNA signals observed in 27.3 % of late 2-cell embryos (n = 21 embryos) with X*ist*^*sense2*^ (green). Signal from the green channel has been amplified to allow visualization of the faint scattered dots.

We then undertook RNA-FISH in embryos originating from crosses identical to those used in our RT-qPCR analysis. In order to locate the *Xist* locus in the nucleus and determine the sex of each embryo, we co-hybridized the *Xist* probe with 2 other probes detecting the expression of the nearby *Rlim* gene and of *Kdm5c*, a gene known to escape from XCI (**Fig. 4A**). In male embryos, we observed a *Xist* pinpoint, indicative of a maternal *Xist* transcription, in ∼20% of nuclei (5 out of 26 blastomeres) of late 2-cell embryos (**Fig. 4A**). This percentage slowly decreased at later developmental stages. In female embryos, on the other hand, we observed, during development, a progressive increase in the number of cells displaying a *Xist* domain with punctuate accumulation of signals already visible in some cells at the late 2-cell stage (**Fig. 4A**). At this stage, however, a majority of cells still showed a single *Xist* pinpoint in agreement with previous reports.^16,60,61^ Only 2 blastomeres out of 22 showed a biallelic signal, indicating a maternal *Xist* transcription.

To increase the RNA-FISH sensitivity and to be able to discriminate *Xist* from *Tsix* transcriptions, we repeated our analysis using strand-specific oligonucleotides probes for *Tsix* and *Xist* on zygotes, early 2-cell embryos, and late 2-cell embryos from crosses involving various genetic backgrounds (C57BL/6 × 129Sv and Pwk × 129Sv-GFP as in the RT-qPCR analysis)(see **Figure S8C** for controls of the specificity of each probe). No significant difference was observed between crosses. In agreement with our RT-qPCR analysis, no *Tsix* transcription could be detected at any stage. In contrast, a *Xist* transcription from the X^M^ was observed in 13% of the male nuclei and a biallelic *Xist* transcription was detected in 20% of female nuclei at the late 2-cell stage (**Fig. 4B**). In order to determine the parental origin of the chromosome transcribing *Xist* in late 2-cell female embryos, we took advantage of the presence of a GFP transgene on the X^P^ in our Pwk × 129Sv-GFP cross.^48^ After sequential DNA-FISH for the transgene and for the *Xic* locus, it appeared that the majority of *Xist* RNA signals co-localized with the GFP signal marking the X^P^. We observed, however, in a small number of nuclei (2 out of 10), biallelic *Xist* expression characterized by a *Xist* pinpoint located at the *Xic* at the vicinity of the GFP marked X^P^ and a second *Xist* pinpoint at the *Xic* located away from the GFP locus (**Fig. 4B**). This indicates that *Xist* may be transiently transcribed from both Xs at the late 2-cell stage. The percentage of cells of late 2-cell embryos exhibiting ongoing maternal *Xist* transcription as detected by RNA-FISH is, however, lower than the proportion of cells of the late 2-cell embryos, in which significant levels of nascent *Xist* transcripts were measured by RT-qPCR (**Fig. 3B**). This difference may indicate that a fraction of nascent *Xist* RNAs and/or introns are exported away from the transcription site to be processed or degraded. Indeed, the high sensitivity of RNA-FISH using fluorescent oligonucleotide probes^62^ allowed us to observe, with the *Xist* intronic probe (*Xist*^*sense2*^), pinpoint signals scattered in the nuclear space in 27.3% of the late 2-cell embryos we analyzed (**Fig. 4C**). This strongly suggests that some blastomeres undergo, at the late 2-cell stage, a burst of *Xist* transcription leading to the production of unstable *Xist* transcripts that are not retained at the transcription site.

## Discussion

Our observation that *Xist* is transcribed during oocyte growth and that this maternal transcription may be transiently re-initiated in late 2-cell embryos suggests that the X^M^ imprint does not act through a strong repression of the maternal *Xist* allele during the first cleavages of the mouse embryo. Physiologically, the late 2-cell stage corresponds to a critical transition in development during which the embryo stops using the maternal products provided by the oocyte and activates the transcription of its own genome. At this time, the genome may be more susceptible to global, less-regulated transcription. Such a hypothesis would suggest that the furtive maternal *Xist* expression we have observed would result from a “leakage” of the *Xist* promoter and lead to the production of non-effective transcripts that are promptly degraded. Indeed, we observed, in some blastomeres of late 2-cell embryos, some *Xist* transcripts away from their transcription site. The maternal *Xist* allele would subsequently and selectively be turned-off at the end of the 2-cell stage suggesting that the maternal imprinting is operational at this stage and is involved in this secondary repression. Alternatively this transient maternal expression of *Xist* at the late 2-cell stage may participate in eliciting inactivation of the X^M^ at later developmental stages in embryos lacking a paternal contribution.^1,4^ Finally, we cannot formally exclude the possibility that the burst of maternal *Xist* RNAs we observe in some late 2-cell embryos is specific of the 3 crosses we have tested (i.e., C57BL/6 x 129Sv, 129Sv x Pwk and Pwk x 129Sv).

A major finding of our study is that *Xist* is significantly induced in early-growing oocytes and that this *Xist* transcription frequently occurs simultaneously with and from the same chromatid as antisense *Tsix* transcription. Interestingly, simultaneous antagonist transcriptions also characterize sense/antisense counterparts involved in the regulation of imprinting at other autosomal loci. In the latter cases, this unusual transcription feature is associated with differential histone modification states and with allele-specific DNA methylation.^45,47^ Intriguingly, however, the offspring of Dnmt3L-deficient females sometimes show normal maternal DNA methylation at autosomal imprinting control regions leaving open the possibility that oocyte-derived marks other than DNA methylation can be recognized in the early embryo.^63^ This parallel suggests that, while the widespread nature of mono-directional transcription throughout the genome does not permit such transcription to be used to specifically mark imprinted loci in early-growing oocytes, overlapping bi-directional transcription could be exploited by the cell to distinguish imprinted from non-imprinted loci. This opens up the possibility that this sense/antisense co-transcription may participate in the initial phase(s) of X^M^-imprinting either via direct recruitment of chromatin remodelers or, indirectly, via the triggering the formation of endo-siRNAs^64^ and subsequent epigenetic modifications at the maternal *Xist*/*Tsix* locus. In this regard, a recent study reported a significant derepression of maternal *Xist* allele upon expression of the H3K9me3 demethylase KDM4B in early parthenogenote embryos, suggesting that this histone mark participates in X^M^ imprinting both by preventing the binding of RLIM on the maternal *Xist* promoter and by precluding maternal *Xist* activation.^65^ H3K9me3 may therefore constitute one of the modifications that are established at the *Xist* promoter during oogenesis concomitantly with *Xist/Tsix* co-transcription. From a mechanistic point of view, concomitant sense/antisense transcription should trigger RNA polymerase II (RNAP II) collision, followed by RNAP II stopping, which, in turn, may lead to primary transcripts remaining associated with the locus for longer periods of time than in the case of a fully processive enzyme event.^66^

Abrogating either *Tsix* or *Xist* transcription during oocyte growth using classical cre-inducible deletions appeared to be impossible due to allele incompatibilities. Notably, we were unable to create the mother that would produce Δ*Xist* oocytes because of an incompatibility between the ZP3-cre inducer allele and the *Xist* floxed allele during spermatogenesis (data not shown). Functional analyses addressing the role of antagonist *Xist* and *Tsix* transcriptions will therefore require developing alternative approaches such as *in vitro* models of oogenesis allowing for *ex vivo* mutagenesis.
