# Supplementary material for: Antagonist Xist and Tsix co-transcription during mouse oogenesis and maternal Xist expression during pre-implantation development calls into question the nature of the maternal imprint on the X chromosome
Source: Epigenetics. 2015 Aug 12;10(10):931–42. doi: 10.1080/15592294.2015.1081327 (PMC4844198; doi:10.1080/15592294.2015.1081327)
Supplement: 1081327_Supplemental_Material.zip [file kepi-10-10-1081327-s001.zip › 1081327_Supplemental Material.docx]

**Supplementary Figure legends**

Figure S1. Controls for the lack of PCR amplification bias in single-cell RT-qPCR analyses.

A. Standard curves of three different target RNAs (*Rplp0*, *Atp7a* and *Gapdh*) after 15, 18 or 20 cycles of pre-amplification of single ES cell cDNAs. Similar slopes of linear regressions are observed independently of the number of cDNA pre-amplification cycles indicating an absence of bias associated with pre-amplification of the target cDNA. All the qPCR assays used in this study have been verified using the same approach.

B. Standard curves of the level of target transcript measured using Biomark qPCR in function of the expected amount of target amplicons after 15, 18 or 20 cycles of single ES cell cDNA pre-amplification. The quantity of cDNA measured using Biomark qPCR after 15, 18 or 20 cycles of single-cell cDNA pre-amplification remains proportional to the initial amount of input target cDNA (curve slope ∼ 2 indicating a linear doubling of target amplicon during the pre-amplification phase).

C. The allelic ratio of *Xist* expression measured on increasing dilutions of single female somatic cell cDNA is not significantly modified after the PCR amplification procedure.

Figure S2. Oocyte quality controls.

A. Quantifications of housekeeping RNAs (*Gapdh*, *Rplp0* and *Hist2h2a*) in the single oocytes shown in **Figure 1C** and **1D** using the Biomark technology. Note that all three RNAs are co-detected in the vast majority of oocytes. The level of variability in the absolute quantifications probably reflects RNA transcription, processing and stability that may slightly differ from one oocyte to the other depending on the RNA metabolism at the time of oocyte collection.

B. Quantifications of RNAs expressed during oogenesis (*Pou5f1*, *Gdf9, Bmp15* and *Zp3*)[^1^](#_ENREF_1) in the single oocytes shown in **Figure 1C** and **1D** using the Biomark technology. As expected all four transcripts show high steady-state levels at all stages of oogenesis. For comparison *Sox2* RNA is less abundant.

C. Hierarchical clustering of gene expression data in oocytes at different stages. Intronic assays are indicated. Other PCR assays amplify exonic products.

Figure S3. Quantification of spliced *Xist* transcripts in MII oocytes of different genetic backgrounds.

Box-plots showing the distributions of spliced *Xist* RNA levels in individual MII oocytes of 129Sv, of PWK/PhJ or of C57/BL6 mouse line. The number of oocytes analysed is indicated for each genetic background. Significant differences in the distributions of transcript level between oocyte populations are indicated underneath each box-plots by *, *t*-test p-values < 0,01; by **, *t*-test p-values < 0,001.

Figure S4. Transcription analysis at the *Xist/Tsix* locus in early growing oocytes.

Histograms showing the absolute RNA levels measured using single-cell RT-qPCR at the indicated positions on 15 freshly-collected early-growing oocytes. Above the histograms, the map shows the reciprocal structures of the *Xist* and *Tsix* transcripts. The positions of the RT-qPCR assays used to detect *Tsix* transcription and primary/spliced *Xist* transcripts are shown as solid bars above and underneath the map respectively. The asterix mark the three assays used in **Figure 1C**. The horizontal dotted line on the histogram marks the average level of spliced *Xist* RNA on the inactive X-chromosome in female somatic cells. No significant differences between transcript levels measured with *Tsix* specific assays, between transcript levels measured with *Xist* intronic specific assays, or between transcript levels measured with *Xist* exonic assays could be detected (*p*>0.05 by χ^2^ test).

Figure S5: Single-cell strand-specific approach.

A. General experimental design. The content of DnaseI treated individual cells is first split into two strand-specific reactions, reverse-transcribing respectively the positive (reverse primer pool) or the negative (forward primer pool) strand. Two control genes (one housekeeping gene *Hist2h2a* and one cell-type specific gene) are systematically included which serve as control for RT efficiency and also control for strand specificity. cDNAs are then pre-amplified through addition of the reciprocal primer either the forward primer in (+)strand reactions or the reverse primer in the (-)strand reactions. ExoI treatment is then applied to remove primer excess and nested assays are used for final RNA quantifications.

B. We initially set-up the strand-specific single-cell procedure on ES cells. Histogram showing RNA levels at the *Sox2* (ES specific gene) and at the *Hist2h2a* (house-keeping gene) loci. As expected significant levels of *Hist2h2a* transcripts were detected in most ES cells and significant levels of both *Hist2h2a* transcripts and of *Sox2* transcripts were detected only in (+)strand reactions and not in (-)strand reactions thereby confirming strand specificity. Reactions without RT (w/o RT) and reactions with RT but omitting RT primers (w/o RT primers) have been run in parallel on a similar batch of individual cells. No amplification was detected in these negative controls. Above the histogram the UCSC screenshot shows the orientation of transcription at each loci.

Figure S6: Strand-specific analysis of transcription at the *Xist* locus in early-growing oocytes.

A. Histogram showing the RNA levels at control genes *Hist2h2a* (house-keeping gene) and *Lamp2* (X-linked gene expressed in oocytes) in 21 individual early-growing oocytes. As expected significant levels of transcripts were detected in (+)strand reactions and not in (-)strand reaction at the *Hist2h2a* loci and vice-versa at the *Lamp2* locus thereby confirming strand specificity. Above each histogram the UCSC screenshot shows the orientation of transcription at the corresponding locus.

B. Histograms showing RNA levels within *Xist* intron 1 on the same batch of 21 early-growing oocytes as in panel A. Upper histogram: 0 to 10^-3^ scale, lower histogram: 0 to 3x10^-5^ scale. High levels of transcripts in *Xist* orientation are detected in the majority of oocytes while concomitant *Tsix* transcription is detected in 5 of these oocytes.

Figure S7: Transcription analysis at the *Igf2r/Airn* locus during oocyte growth.

A) Map of *Igf2r/Airn* locus showing the positions of *Igf2r* and *Airn* PCR assays. Grey boxes: exons.

B) Histograms showing the steady-state level of *Airn* and *Igf2r* RNAs in early-growing MI oocytes, in late-growing NSN and SN MI oocytes and in MII oocytes.

Figure S8: Transcription and nuclear organisation at the *Xic* in early-growing MI oocytes.

A. Representative image showing the maximal projections of the early-growing MI oocyte after RNA-FISH shown in **Figure 2A** (left panel) and subsequent DNA FISH (right panel). Probes used for hybridisations are indicated on the scheme above the pictures. Magnifications of nucleus area around the signals are shown.

B. RNA-FISH analysis on female ES cells with fluorescent oligonucleotide probes detecting either *Xist* or *Tsix* as depicted on the scheme above the RNA-FISH images. The Table beside indicates the percentage of chromosome showing a *Tsix*-specific, a *Xist*-specific or a co-expression of both transcripts in ES cells (n=145 nuclei). RNA-FISH on somatic female cells with the same probe combination assess the strand-specificity of *Xist* and *Tsix* probes.

C. Control for the specificity of oligonucleotide probes used in **Figure 4**. RNA-FISH analysis on differentiated female cells with fluorescent oligonucleotide probes detecting either *Xist* nascent transcripts (*Xist^sense2^*) or *Tsix* antisense transcription (*Tsix^antisense^*) and with double-stranded probe detecting the transcription at the *Xist* locus (both sense and antisense) and detecting *Xist* mature transcripts (p510 probe) as depicted on the scheme above the RNA-FISH images. As expected in differentiated female cells the p510 probe detects both *Xist* active transcription site and *Xist* spliced RNAs coating the X-chromosome, while *Xist* intronic oligonucleotides (*Xist^sense2^*) only detects *Xist* transcription site. In these cells no *Tsix* transcription is detected in agreement with the lack of antisense expression at this stage. In contrast active antisense transcription is observed in undifferentiated ES cells. Therefore this combination of probes allows discriminating ongoing *Xist* transcription from ongoing *Tsix* transcription and from *Xist* maturing RNAs.

Figure S9: Quantification of poly(A)+ *Rlim* and *Xist* transcripts in MII oocytes.

Pie charts showing the relative amounts of *Xist* and *Rlim* poly(A)+ mature transcripts in a pool of ~1000 129Sv MII oocytes that have been pulled down on oligo(dT) columns. Drosophila poly(A)+ RNA, *Taf11,* serves as poly(A)+ control and as carrier RNA. The vast majority of *Xist* (87%) and *Rlim* (91%) RNAs present in MII oocytes bear short poly(A) tails that do not bind to the oligo(dT) column.

Figure S10: Allele-specific RT-qPCR analysis of transcription at the *Xist/Tsix* locus in 129Sv/Pwk preimplantation embryos.

(A) Box-plots showing the distribution of transcript levels in whole female and male embryos obtained from a (Pwk x 129Sv) cross assessed by RT-qPCR using the Biomark technology. Tsix, Xist IN, and Xist Trans-EX PCR assays are the same as in **Figure 1C** except that allelic assays have been used here (see **Table S1** for primer sequence). 2-cell (E): early 2-cell embryos (female, n=3; male, n=3); 2-cell (L): late 2-cell embryos (female, n=3; male, n=10); 4-cell: 4-cell embryos (female, n=9; male, n=14); 8-cell: 8-cell embryos (female, n=12; male n=5); M: morulae (female, n=6; male, n=3). See method section for embryo sexing. The levels of maternal, intronic, *Xist* transcripts in late 2-cell embryos are significantly different from the levels of maternal, intronic, *Xist* transcripts in early 2-cell or in 4-cell embryos in both male and female (*p*<0.05 by KS test).

(B) Scatter-plots of *Tsix* expression levels from the paternal (x-axis) relative to the maternal (y-axis) X chromosome measured with *Tsix* RT-qPCR assay (**Figure 1C**) on the same batch of individual cells as in **Figure 3C**. Each dot represents a cell.

(C) Distribution of the level of indicated transcripts in individual cells of (129Sv/Pwk) male embryos at the indicate stage as measured by single cell RT-qPCR using assays shown in **Figure 1C**. Each dot represents a cell. A significant difference in *Xist* expression is detected with Xist IN in cells of late 2-cell embryos as compared to cells of embryos at either earlier or later stages of development (*p*<0.05 by KS test).

**Supplementary Figure legend references**

1. Hamatani T, Yamada M, Akutsu H, Kuji N, Mochimaru Y, Takano M, Toyoda M, Miyado K, Umezawa A, Yoshimura Y. What can we learn from gene expression profiling of mouse oocytes? Reproduction 2008; 135:581-92.
