# Supplementary figures and images for: Antagonist Xist and Tsix co-transcription during mouse oogenesis and maternal Xist expression during pre-implantation development calls into question the nature of the maternal imprint on the X chromosome
Source: Epigenetics. 2015 Aug 12;10(10):931–42. doi: 10.1080/15592294.2015.1081327 (PMC4844198; doi:10.1080/15592294.2015.1081327)

A

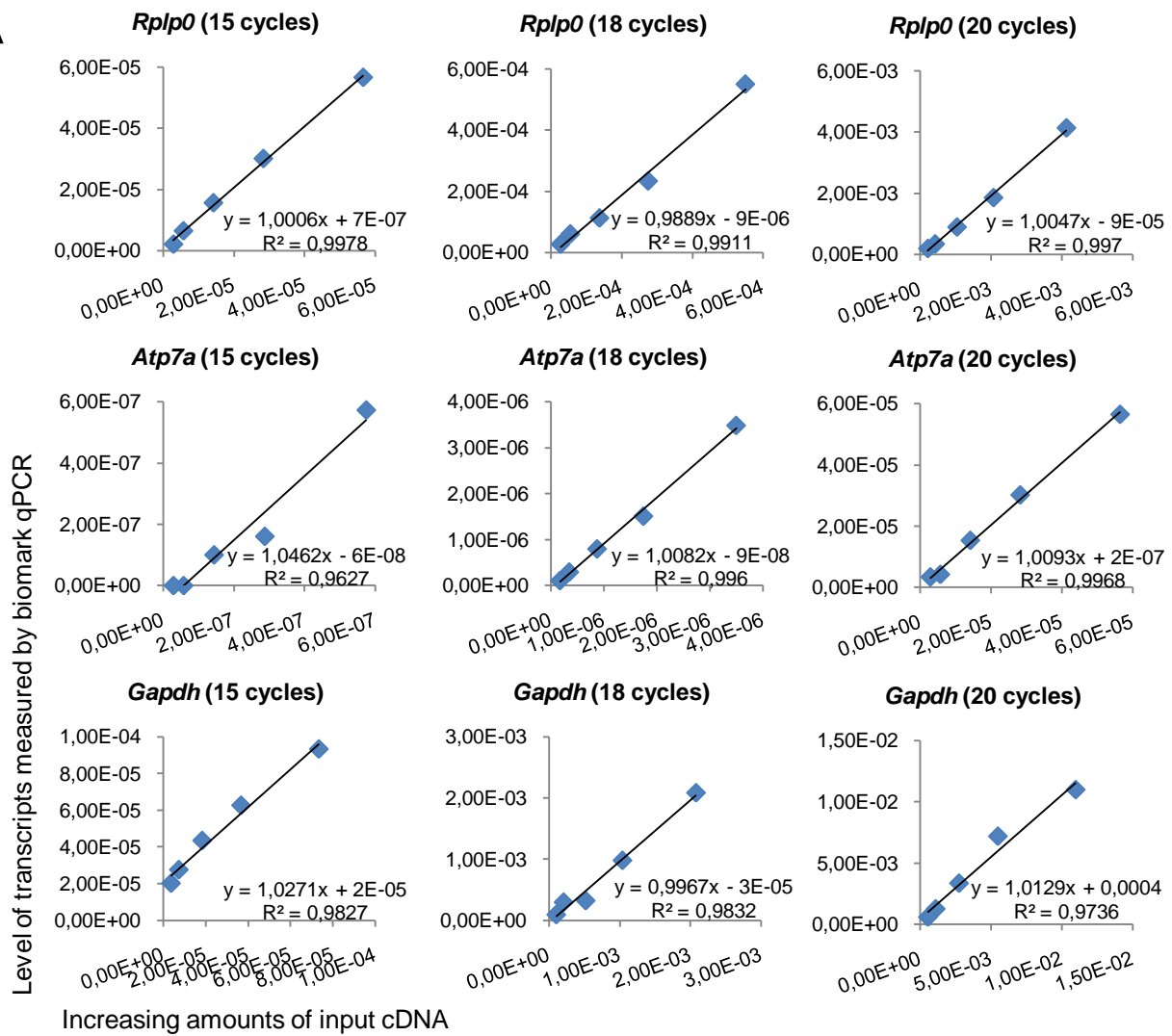

B

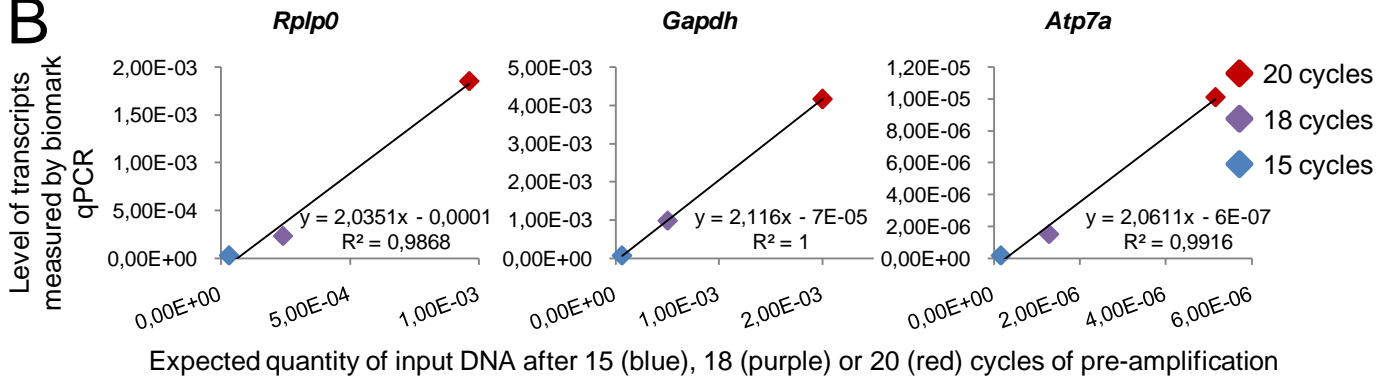

C

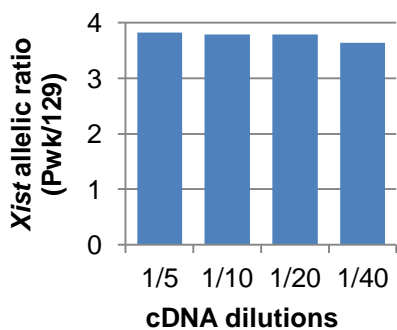

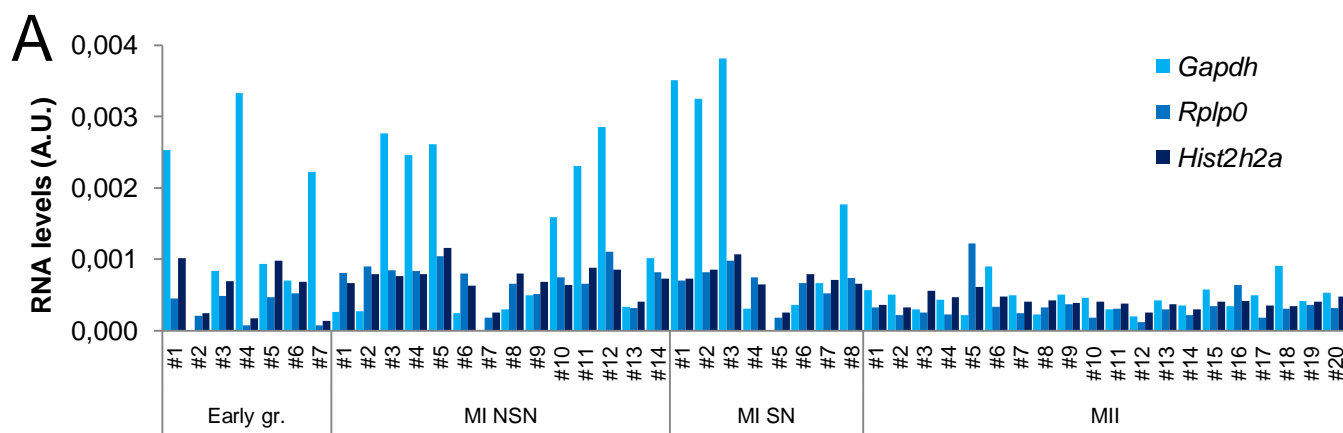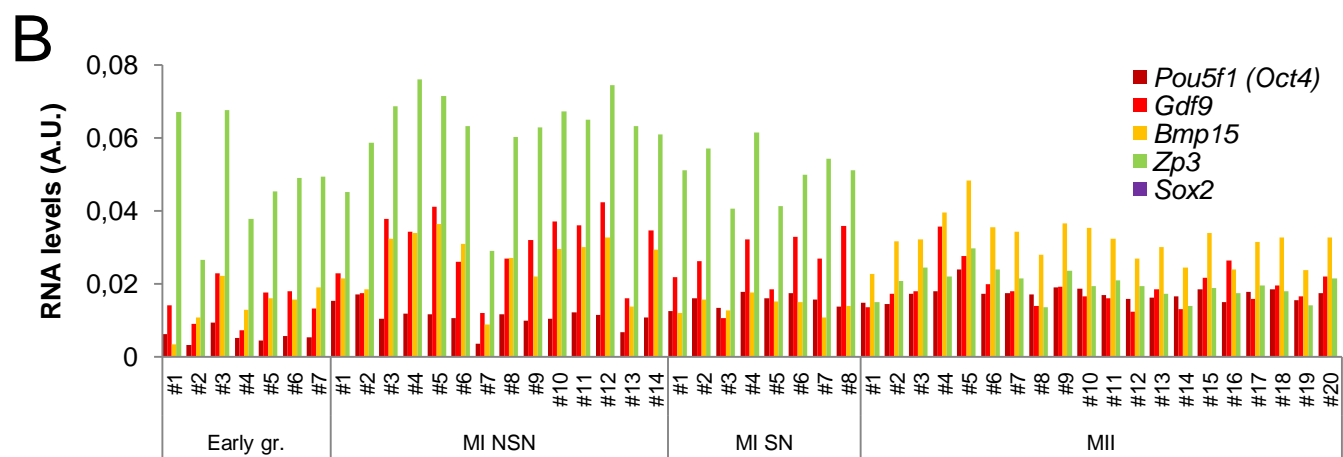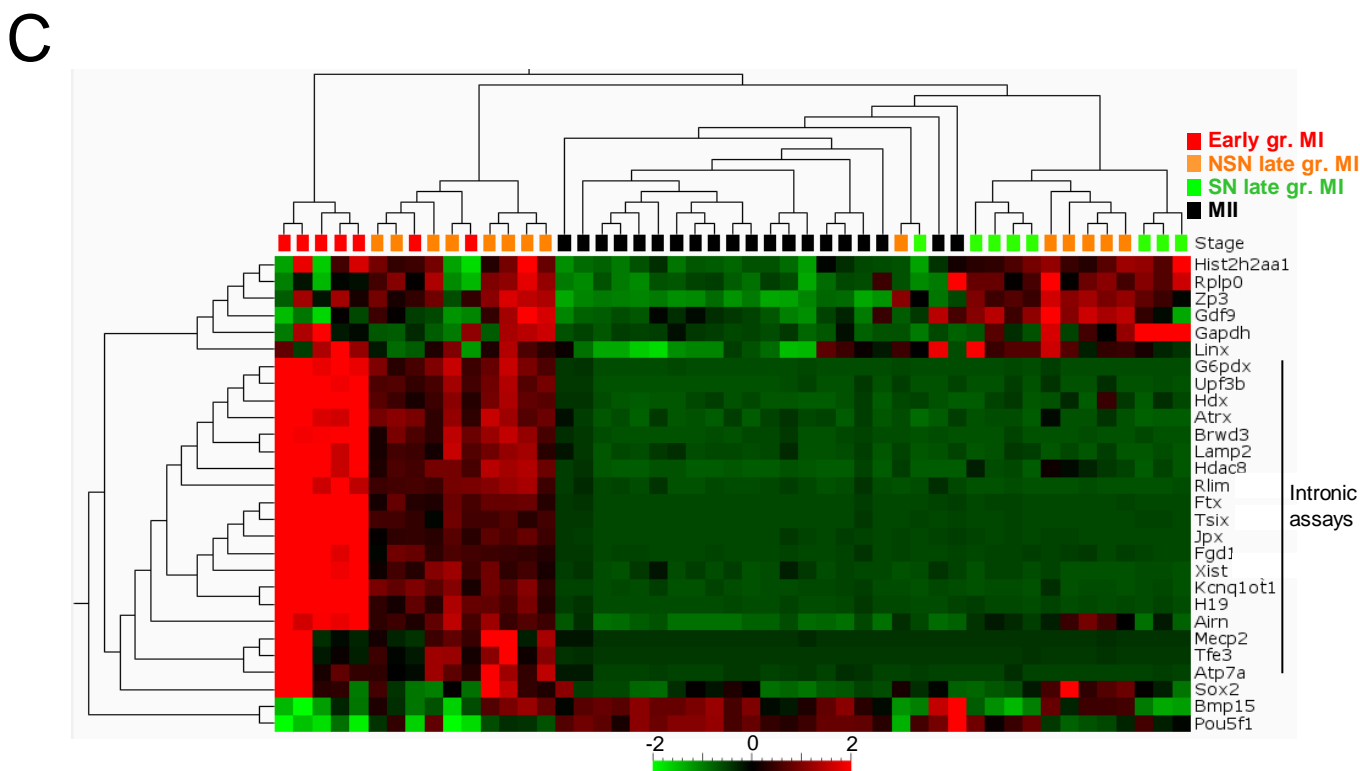

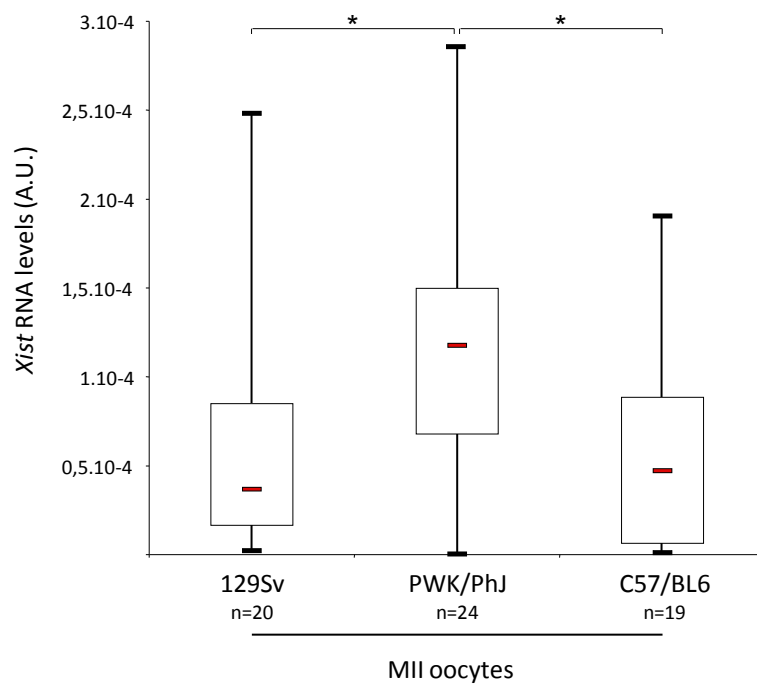

Sup 3

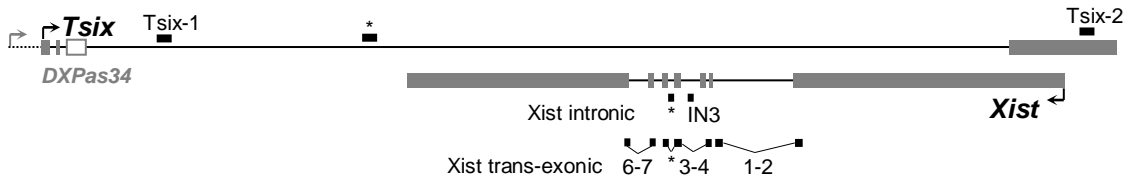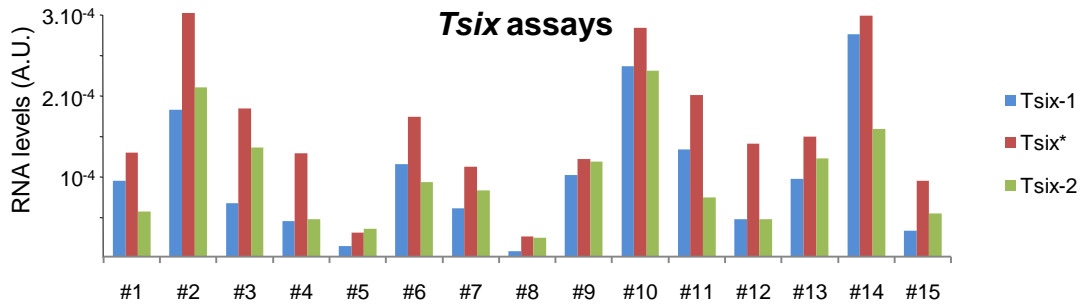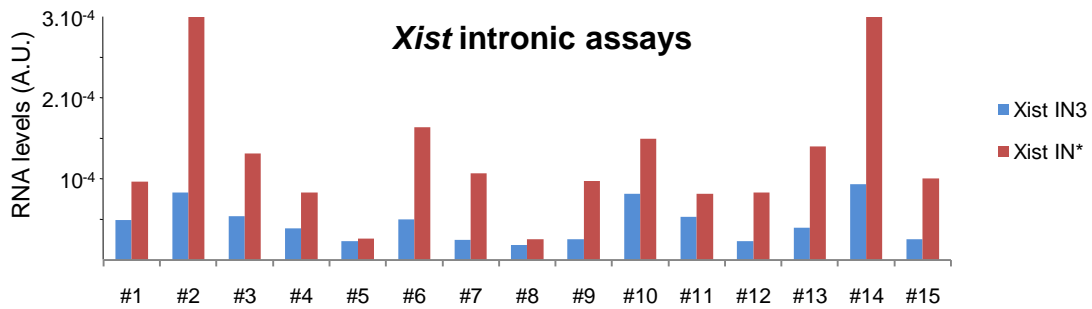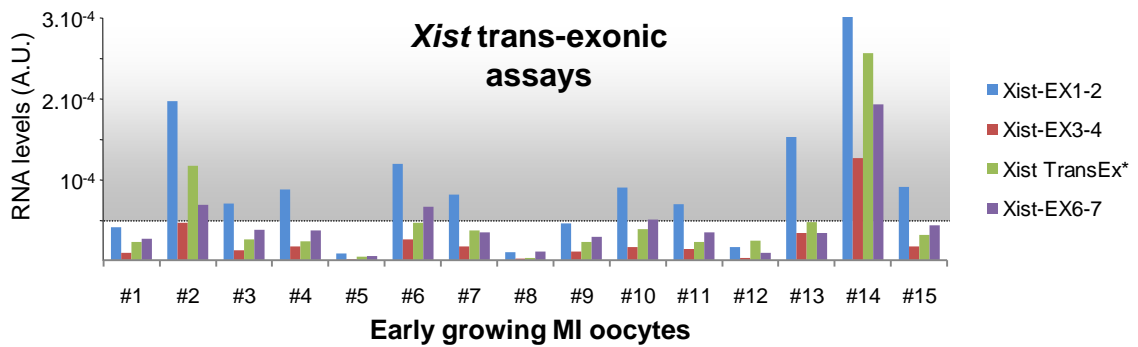

**A**

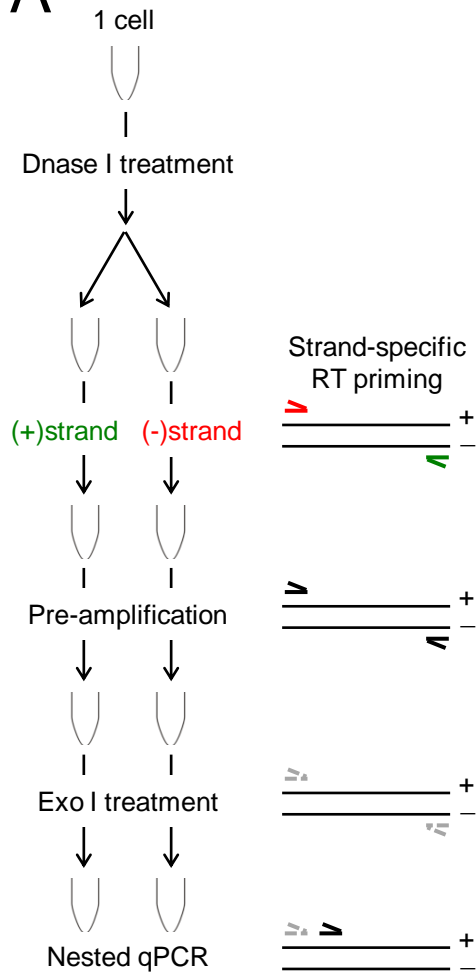

**B**

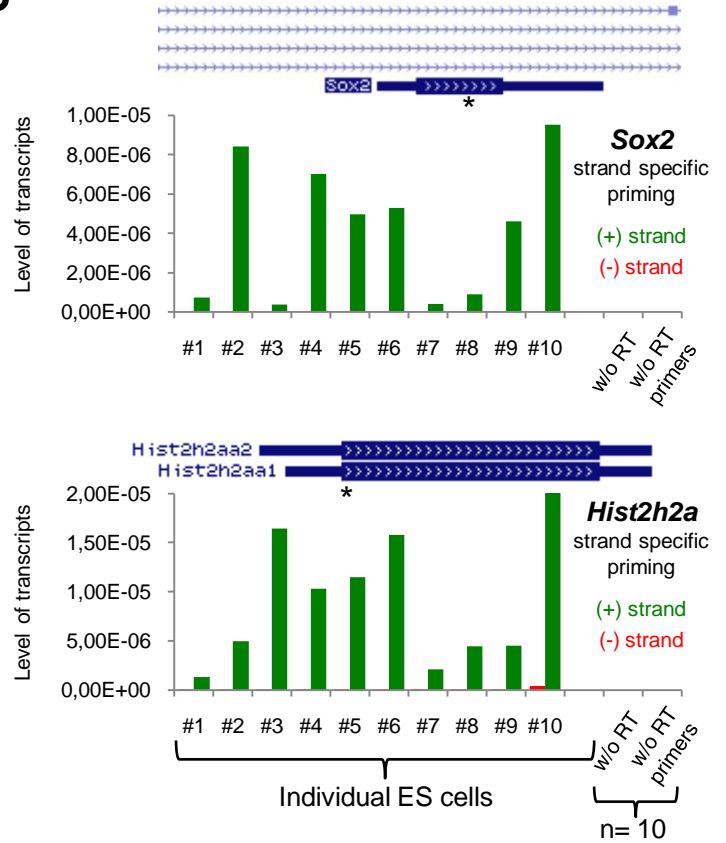

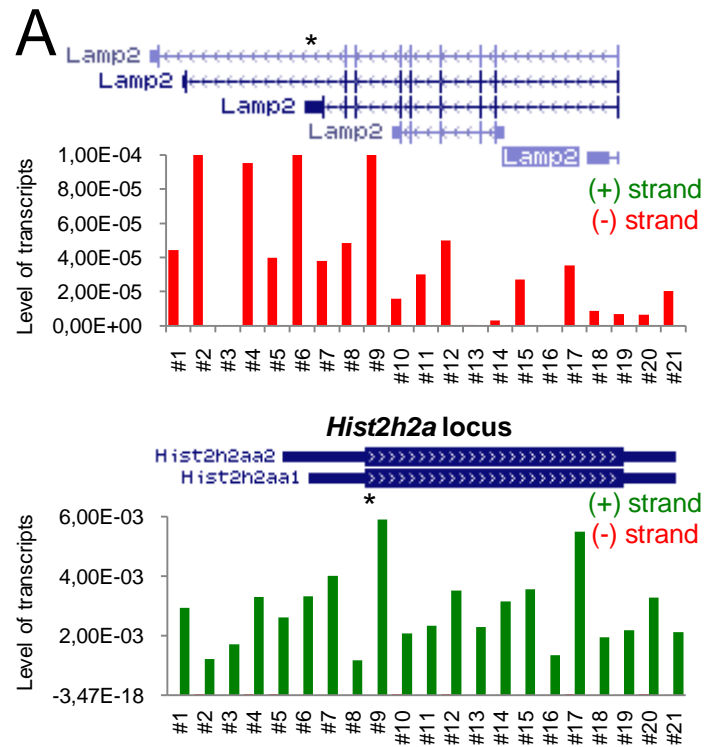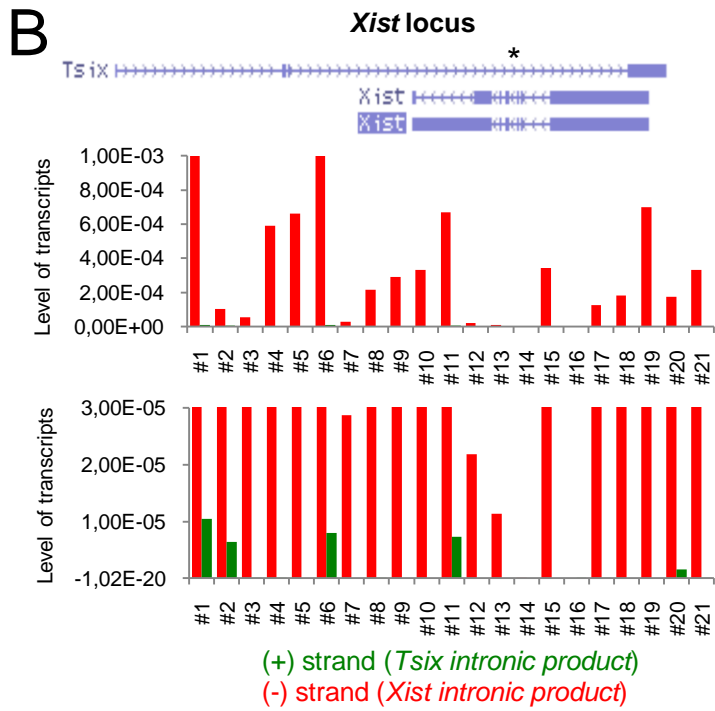

[illegible]

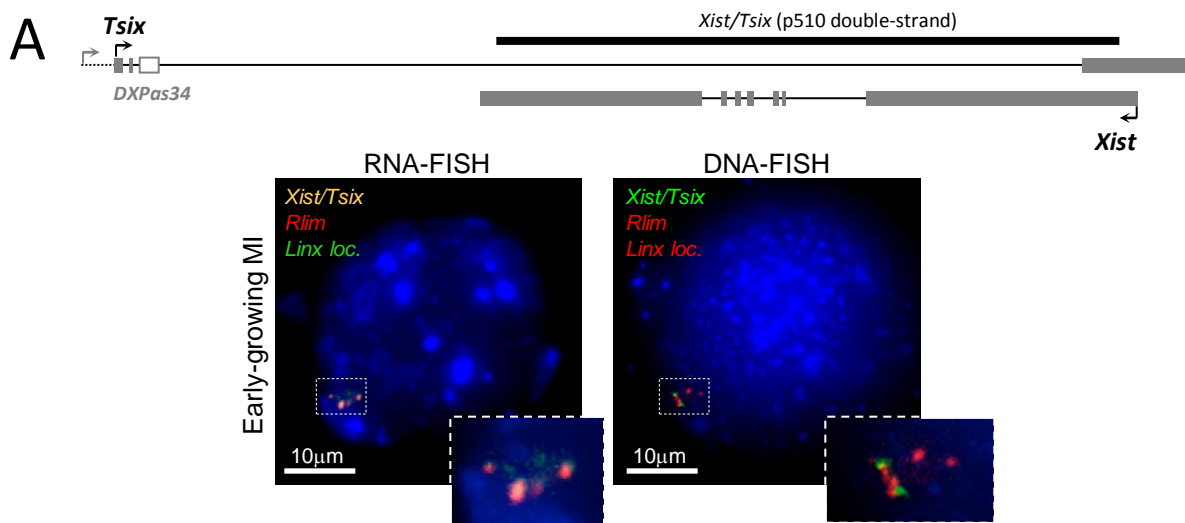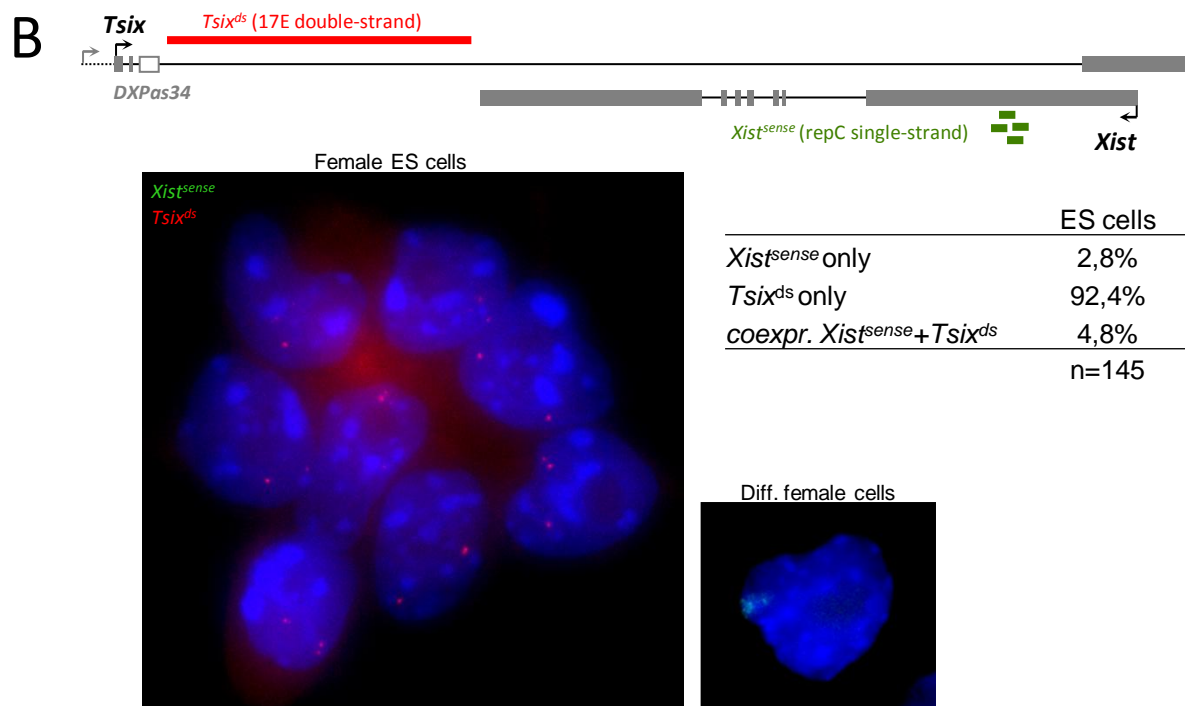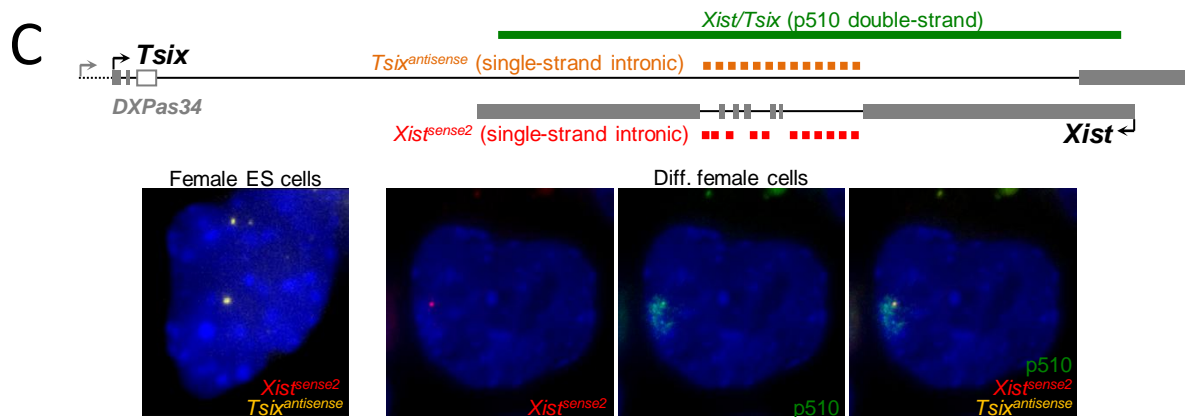

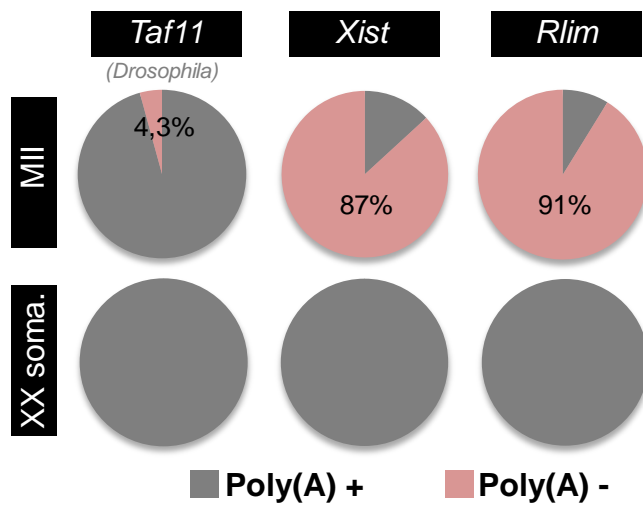

Sup 9

# A

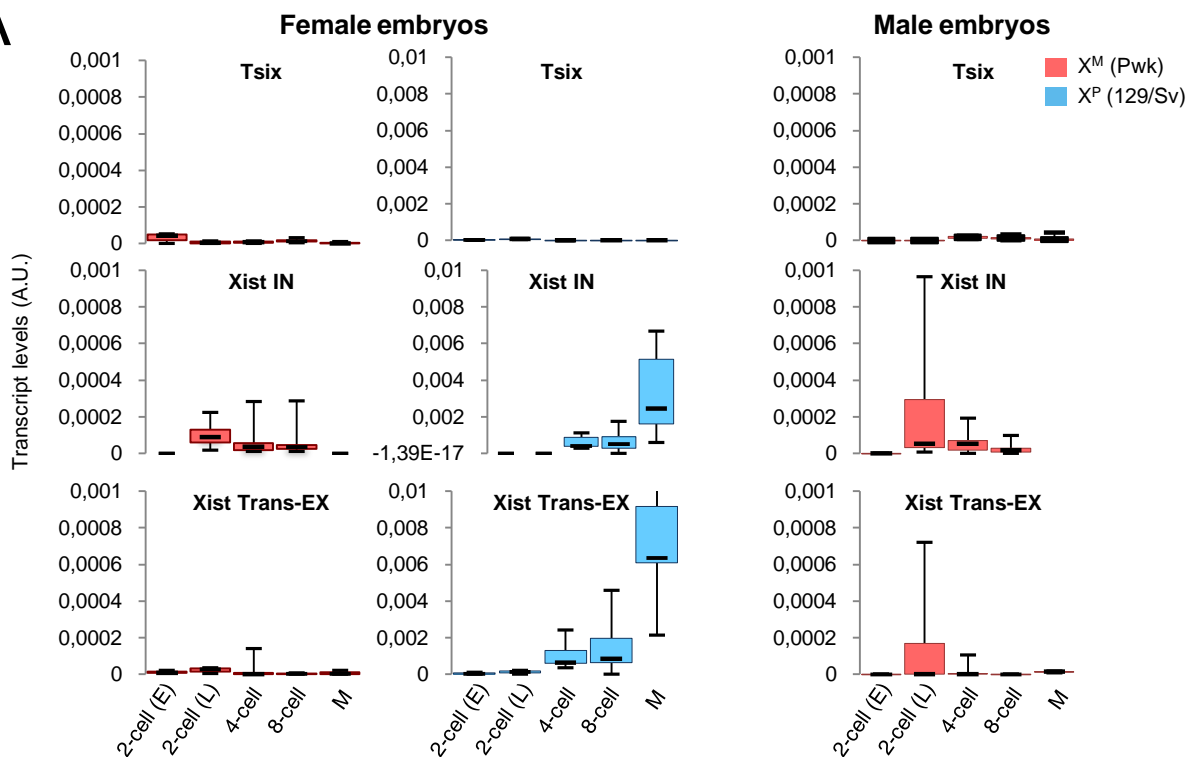

# B

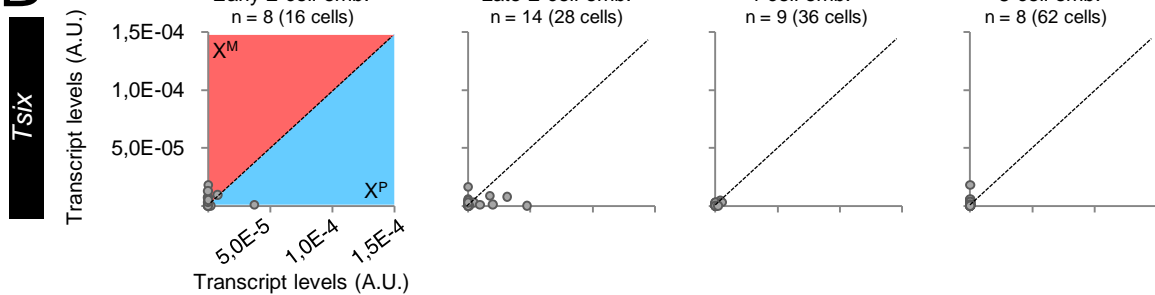

# C

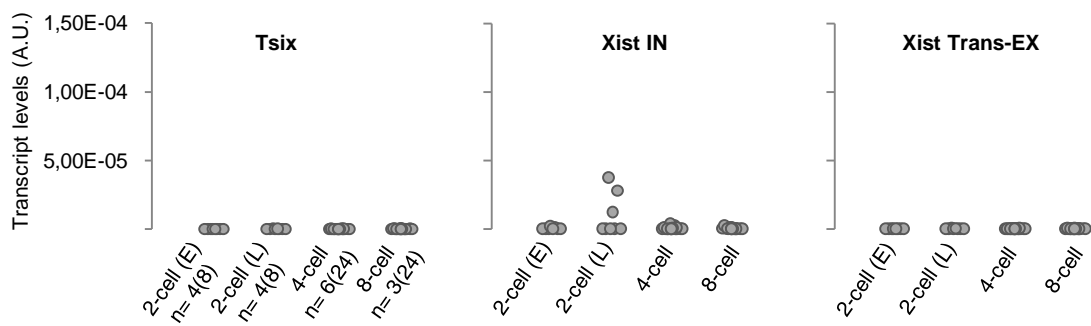

Supplement: 1081327_Supplemental_Material.zip [file kepi-10-10-1081327-s001.zip › Supplemental Figures.pdf]
